# Assessment of 10CrMo9-10 Power Engineering Steel Degradation State by Using Small Punch Test

**DOI:** 10.3390/ma18174133

**Published:** 2025-09-03

**Authors:** Kamil Majchrowicz, Barbara Romelczyk-Baishya, Monika Wieczorek-Czarnocka, Szymon Marciniak, Milena Mras, Dominik Kukla, Mateusz Kopec, Zbigniew Pakieła

**Affiliations:** 1 Faculty of Materials Science and Engineering, Warsaw University of Technology, Wołoska 141, 02-507 Warsaw, Poland; barbara.romelczyk-baishya@pw.edu.pl (B.R.-B.); monika.wieczorek-czarnocka@pw.edu.pl (M.W.-C.); szymon.marciniak@pw.edu.pl (S.M.); milena.mras.stud@pw.edu.pl (M.M.); zbigniew.pakiela@pw.edu.pl (Z.P.); 2 Institute of Fundamental Technological Research, Polish Academy of Sciences, Pawińskiego 5b, 02-106 Warsaw, Poland; dkukla@ippt.pan.pl (D.K.); mkopec@ippt.pan.pl (M.K.); 3 College of Science and Engineering, University of Derby, Markeaton Street, Derby DE22 3AW, UK

**Keywords:** 10CrMo9-10 steel, small punch test, mechanical properties, degradation

## Abstract

Degradation of power engineering steel structures requires constant monitoring of their mechanical properties to estimate remaining service life. Therefore, the current study aimed to develop a methodology that will enable for accurate determination of changes in mechanical properties of 10CrMo9-10 steel after long-term exploitation involving the Small Punch Test (SPT). Firstly, the as-received 10CrMo9-10 steel was annealed at 770 °C for different periods (1.5, 6 and 24 h) to deteriorate its strength to a level similar to its exploited counterpart. Then, mechanical properties were characterized by uniaxial tensile tests and the SPT method using miniaturized discs with a diameter of 8 mm and a thickness of 0.5 mm as recommended by the EN 10371:2021 standard. It allowed to determine a formula correlating the SPT results (i.e., elastic–plastic transition force and maximum force) with the yield and ultimate tensile strength. The *β_Rp_*_0.2_ and *β_Rm_* correlation factors were equal to 0.437 and 0.255, respectively. Finally, the exploited 10CrMo9-10 steel was tested by the SPT method. Based on the SPT results, the values of *R_p_*_0.2_ = 236 ± 27 MPa and *R_m_* = 459 ± 17 MPa were estimated, which were close to those assessed during the uniaxial tensile tests (*R_p_*_0.2_ = 218 ± 3 MPa and *R_m_* = 454 ± 4 MPa). It was shown that the application of such a relatively simple method is a promising way for determining the changes in mechanical properties of structural steels after long-term service at elevated temperature.

## 1. Introduction

10CrMo9-10 (also known as 10H2M or 1.7380) is a chrome-molybdenum-manganese, creep-resistant steel which is widely used in thermal power plants due to its ability to withstand long-term service under elevated temperature and high pressure [1,2,3]. Typically, such Cr-Mo-Mn steels are intended for boilers and steam turbine components operating at temperatures up to 580 °C [3,4]. The addition of Cr enhances strength, hardness and corrosion and oxidation resistance. Molybdenum is particularly effective at improving strength and impact toughness, while Mn is a key element for increasing weldability and ductility [5,6]. Moreover, Cr, Mo and Mn significantly enhance hardenability and promote the formation of uniform and fine microstructures, such as bainitic and martensitic, with numerous carbide precipitates [5,6]. Depending on the applied normalizing and tempering or quenching and tempering parameters, 10CrMo9-10 steel may exhibit a martensitic, bainitic or mixed bainitic–ferritic microstructure [3,7,8]. The microstructure type is controlled by the applied cooling rate, with faster cooling favouring martensite formation [6].

The degradation of structural steels in thermal power plants is related to different damage mechanisms, such as creep damage, high temperature fatigue, thermal ageing and microstructural degradation leading to embrittlement, hydrogen damage, erosion or high temperature corrosion and oxidation [9]. Cr-Mo steels are mainly susceptible to microstructural degradation due to coarsening of carbides or impurity segregation at grain boundaries [10,11]. During long-term exposure at elevated temperature, the microstructure degradation process results in the coarsening of martensite or bainite laths into broader ferrite laths and subsequently into equiaxed ferrite grains. Additionally, the coarsening of M_2_X carbides into larger M_23_C_6_ and/or M_6_C carbides occurs, leading to the formation of an equiaxed ferrite grain structure with the coarse carbides concentrated on the ferrite grain boundaries [1,2,7]. Such microstructure reorganization significantly deteriorates the mechanical properties of 10CrMo9-10 steel components [2,7]. This implies a need for their constant monitoring through a series of non-destructive and destructive tests [1]. The assessment of the current condition of steel is mainly realized by a comparative study of the as-received and exploited materials with standard requirements [12,13]. It mainly covers the characterization of microstructural changes and mechanical properties determined during tensile and Charpy tests [7], or additional fatigue [2,13] and creep tests [14]. This approach typically requires a relatively high volume of tested material and a long time of structure shut down in order to perform a complete evaluation of its current condition.

The small punch test (SPT) is a method that enables the characterization of different mechanical properties of small-scale materials with a limited volume [15,16] or materials extracted from in-service components [17]. During the SPT, a disc-shaped or rectangular miniaturized specimen (having a size of a few millimetres) is deformed through a die using a hemispherical punch. Subsequently, the recorded load–deflection data is adopted for the estimation of the tensile strength, fracture toughness or creep properties [15,18]. A successful estimation of a yield strength (*R_p_*_0.2_) and ultimate tensile strength (*R_m_*) [18,19], along with the evaluation of fracture toughness [20,21,22], ductile-to-brittle transition temperatures [23,24] or creep properties [25,26] has been widely reported in recent years. As a consequence, the European Committee for Standardization has recently approved this method in the EN 10371:2021 standard [27]. According to this standard, a linear correlations of the SPT results with the *R_p_*_0.2_ and *R_m_* values were proposed as follows:

(1) $$R_{p0.2}\;=\;\beta_{Rp0.2}\cdot \frac{F_{e}}{{h_{0}}^{2}}$$

(2) $$R_{m}=\beta_{Rm}\cdot \frac{F_{m}}{\left(h_{0}\cdot u_{m}\right)}$$

where *h*_0_ is an initial specimen thickness, *F_e_* is an elastic–plastic transition force in the small punch test, *F_m_* is a maximum force during the test, *u_m_* is a deflection at the maximum force, and *β_Rp_*_0.2_ and *β_Rm_* are correlation factors for estimation of *R_p_*_0.2_ and *R_m_*, respectively.

Kumar et al. [28] reported the *β_Rm_* value of 0.281 based on the SPT results of 20MnMoNi55, CrMoV and SS304LN steels using miniaturized discs with a diameter of 3 mm and a thickness of 0.25 mm punched through a die with a diameter of 1.5 mm. In turn, Garcia et al. [29] used rectangular 10 mm × 10 mm blanks with a thickness of 0.5 mm to investigate a wide range of steels with a different strength, i.e., two experimental grades of Eurofer steel, four grades of vanadium-modified 2.25Cr1Mo steels, automobile dual phase steels, AISI 304 and D2205 stainless steels, and general structural steels such as S460, API X70 and AR. The authors obtained the *β_Rp_*_0.2_ and *β_Rm_* factors of 0.442 and 0.277, respectively, showing that these coefficients are less sensitive to the tested material. Similar findings were reported by Altstadt et al. [30] who used disc-shaped specimens having a diameter of 8 mm and a thickness of 0.5 mm made of P91, P92, Eurofer97, 22NiMoCr37 and 15Kh2MFA steels. The *β_Rm_* factor varied from 0.254 to 0.299 depending on the tested material, with the average value for all materials equal to 0.278. It was concluded that the SPT correlation coefficients are more dependent on the geometry parameters of the testing setup, i.e., the specimen size and thickness, the radius of the punch, a receiving hole diameter or a chamfer size and shape. It has been clearly shown by Altstadt et al. [31] for the T91 steel that the *β_Rp_*_0.2_ and *β_Rm_* factors may differ from 0.42 to 0.63 and 0.19 to 0.26, respectively, depending on the used SPT setup.

Since the SPT proved its efficiency in steel testing, the current study aimed to develop a methodology for determining changes in the mechanical properties of 10CrMo9-10 steel after long-term exploitation (280,000 h) at elevated temperature and internal pressure (540 °C and 2.9 MPa) using the SPT method. Since such steel is one of the most popular steels in the power engineering sector in Poland [32] or Czech Republic [33], the assessment of its mechanical performance after prolonged service is of great importance. In addition, to the best of the authors’ knowledge, no literature references provide the exact correlation factors for estimation of *R_p_*_0.2_ and *R_m_* from the SPT results according the current EN 10371:2021 standard. So far, Andres et al. [33] and Dymacek et al. [34] have focused on the creep properties of the exploited 10CrMo9-10 steel, while Kaliciak et al. [35] did not present any correlation formulas for recalculating the SPT results.

## 2. Materials and Methods

The chemical composition of the investigated 10CrMo9-10 steel in the as-received and exploited state was presented in Table 1 with respect to the EN 10028-2:2017 standard requirements [36]. The exploited material was a part of a pipe having a diameter of about 500 mm and a wall thickness of 20 mm, which operated for 280,000 h at the internal pressure of 2.9 MPa under a temperature of 540 °C. The as-received 10CrMo9-10 steel was cut into cylindrical slabs with a diameter of 60 mm and a thickness of 25 mm and annealed at 770 °C for 1.5, 6 and 24 h to obtain several conditions with gradually deteriorated mechanical properties to the final level similar to its exploited counterpart. Furthermore, such different material states will allow to estimate the linear correlations of the SPT results with the standard tensile parameters (i.e., *R_p_*_0.2_ and *R_m_*). The annealing temperature was established based on the dilatometric measurements performed on a representative sample in the form of a rod with a diameter of 3 mm and a length of 10 mm using a TA Instruments DIL805L (TA Instruments, New Castle, DE, USA) dilatometer at a heating rate of 0.3 °C/min.

The microstructure of 10CrMo9-10 steel was characterized by a Zeiss Axio Observer (Carl Zeiss Microscopy GmbH, Jena, Germany) microscope. Sections for microscopic observations were ground, polished and etched with a solution of 10% nital. The microstructure was quantified in terms of a mean grain boundary density (*S_V_*), carbide precipitate density (*N_A_*) and a fraction of equiaxed ferrite grains (*V_V_*) using an ImageJ software (v1.53k). A line-intercept method was used for the grain boundary density measurement. The Vickers hardness measurements (at least 10 indentations per sample) were conducted at a load of 5 kg using an Innovatest Falcon 500 (Innovatest Europe BV, Maastricht, The Netherlands) hardness tester.

**Figure 1:**
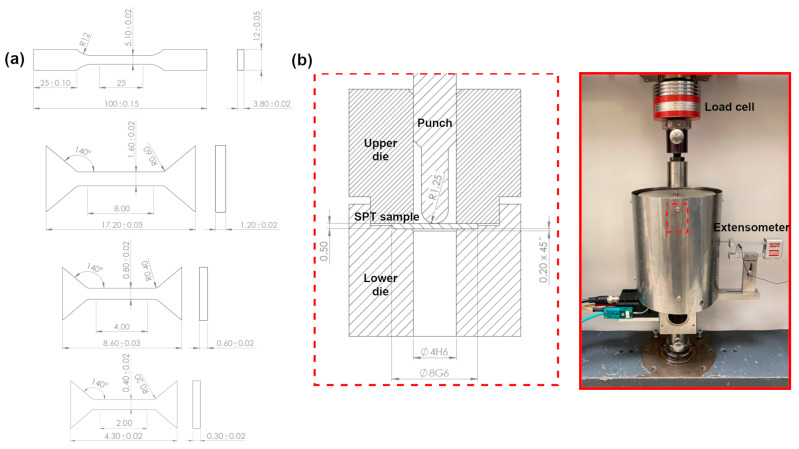
(**a**) Technical drawings of flat standard and miniaturized tensile samples and (**b**) schematic and general view of the SPT experimental setup.

The tensile properties of the as-received state were assessed according to the ISO 6892-1 standard [37] using standard flat samples with a gauge length of 25 mm and a cross section of 3.8 mm × 5.1 mm as well as miniaturized flat samples with the different gauge length of 8, 4 and 2 mm and proportional cross sections (as shown in Figure 1a). Such tests aimed to find an optimal sample geometry to minimize the specimen size which can be directly extracted from in-service components without a need of repair after material removal. A scoop cutter sampling technique can be used for such purposes [27,38]. It is a unique hemispherical shell cutter capable of removing a disc-shaped material volume (typically with a diameter of approximately 40 mm and a thickness of 4 mm) without mechanical distortion or thermal degradation of the component [27,38]. All other states were examined using miniaturized test specimens with a gauge length of 8 mm showing the same results as the standard ones. Uniaxial tensile tests of standard specimens were performed using an MTS 810 (MTS Systems Corp., Eden Prairie, MN, USA) servo-hydraulic testing machine equipped with a 100 kN load cell and an MTS 632.24F-50 extensometer. The miniaturized test specimens were tested using a Zwick Roell Z005 (Zwick GmbH & Co. KG, Ulm, Germany) testing machine with a 5 kN loading capacity and a digital image correlation (DIC) system for strain measurements (described in more detail in [39,40]). All tensile tests were conducted at the initial strain rate of 10^−3^ s^−1^. Each material state was represented by at least five samples cut by the electric discharge machining (EDM) method along the rod or pipe axis. The measurement uncertainties were calculated according to Annex J from the ISO 6892-1 standard as the combined uncertainty of the standard deviations and the accuracy of the measuring equipment, i.e., the load cell (±0.5%), extensometer (±0.5%) or DIC strain measurement system (±1%), gauge length, and cross section measurements (±1%).

The experimental SPT setup consisted of a lower die with a receiving hole diameter of 4 mm and a chamfer of 0.2 mm × 45° and a spherically ended punch having a radius of 1.25 mm as recommended by the EN 10371:2021 standard [27] (Figure 1b). The deflection was measured by an MTS 634.12F-25 extensometer (MTS Systems, Berlin, Germany) attached to the lower surface of the sample. Before the tests, the disc-shaped SPT samples with a diameter of 8 mm were ground using 1200-grit SiC paper to obtain the required thickness of 0.5 ± 0.005 mm. The SPTs were conducted on a Zwick Roell Z005 (Zwick GmbH & Co. KG, Ulm, Germany) testing machine at a crosshead speed of 0.5 mm/min. At least five SPT samples were tested from each material state. The measurement uncertainties of the load and deflection were estimated based on the standard deviations and the equipment errors (i.e., the accuracy of load cell ± 0.5% and extensometer ± 0.5%).

## 3. Results and Discussion

### 3.1. Heat Treatment

**Figure 2:**
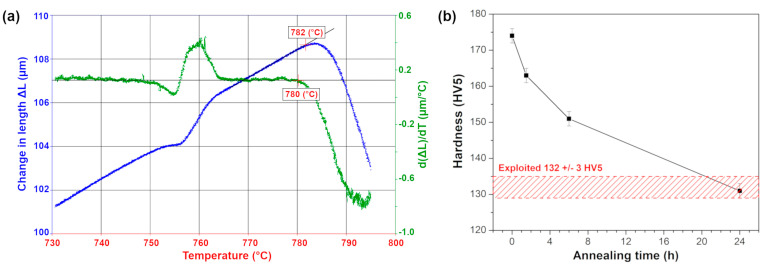
(**a**) Dilatometric curve of as-received 10CrMo9-10 steel and (**b**) Vickers hardness changes as a function of annealing time at 770 °C.

The dilatometric measurements were firstly performed for the as-received 10CrMo9-10 steel in order to determine the maximum heat treatment temperature to avoid austenitic transformation. The measured length change as a function of temperature is presented in Figure 2a. The calculated onset temperature of austenitic transformation was around 781 °C, which is very close to the temperature reported by Ławrynowicz [3], i.e., 780 °C. Thus, the as-received 10CrMo9-10 was annealed at 770 °C for a specified time of 1.5, 6 and 24 h. The changes in the Vickers hardness of the annealed samples are shown in Figure 2b. Hardness gradually decreased from 174 ± 2 HV5 for the as-received state to the value of 131 ± 2 HV5 after 24 h of annealing, which was practically the same as the hardness of the exploited 10CrMo9-10 steel (i.e., 132 ± 3 HV5).

### 3.2. Microstructure

**Figure 3:**
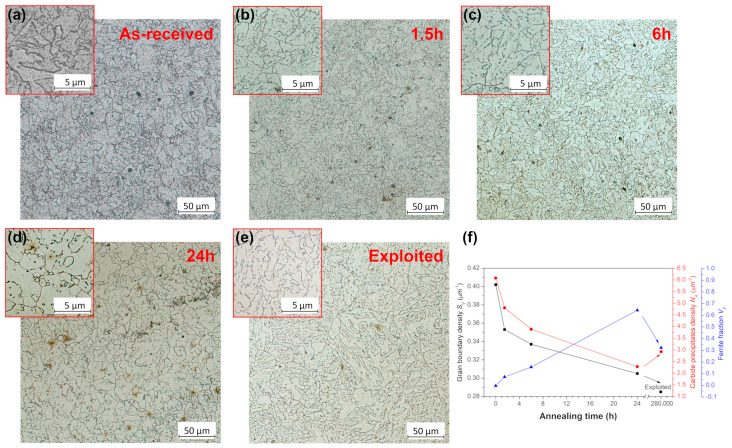
Microstructure of (**a**) as-received, (**b**–**d**) annealed and (**e**) exploited 10CrMo9-10 steel; (**f**) grain boundary–carbide precipitates–ferrite fraction relation as a function of annealing time.

Figure 3a–e presents the microstructure of the 10CrMo9-10 steel in the as-received, annealed and exploited states. The as-received 10CrMo9-10 steel exhibited a fully bainitic microstructure with carbides located at grain boundaries and inside grains, which is commonly observed in this steel grade, as reported by Gwoździk et al. [7] and Wang et al. [8]. During annealing at 770 °C, the bainite laths transformed into broader ones and subsequently into equiaxed ferrite grains, while the observed carbides coarsened and segregated on the ferrite grain boundaries, as presented for the 10CrMo9-10 steel after annealing for 24 h. The calculated grain boundaries density *S_V_* and carbide precipitates density *N_A_* gradually decreased for longer annealing periods, as shown in Figure 3f, whereas the fraction of equiaxed ferrite grains increased and became a dominant constituent after 24 h of annealing. In turn, the microstructure of the exploited state consisted of a mixture of degraded bainite and a lower content of ferrite grains with numerous carbides concentrated at grain boundaries. Similar microstructural changes during long-term exposure at elevated temperature of 10CrMo9-10 steel were reported earlier by Brodecki et al. [2], Gwoździk et al. [7] or Kopec et al. [41], which resulted in a significant deterioration of its mechanical performance [2,7,41].

### 3.3. Tensile Properties

**Figure 4:**
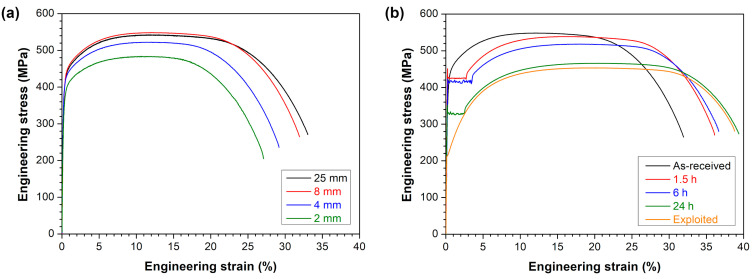
Representative stress–strain curves of 10CrMo9-10 steel in: (**a**) as-received state for different specimen size, (**b**) as-received, annealed and exploited conditions.

The effect of specimen geometry on the tensile properties of the as-received 10CrMo9-10 steel is presented in Figure 4a and Table 2. The following mechanical parameters were achieved for the as-received 10CrMo9-10 steel tested by the standard size samples: the 0.2% offset yield strength *R_p_*_0.2_ = 407 ± 11 MPa, the ultimate tensile strength *R_m_* = 538 ± 6 MPa, the uniform elongation *A_u_* = 11.7 ± 0.2% and the elongation at break *A* = 32.1 ± 1.9%. The miniaturized samples with the gauge length of 8 mm showed very similar mechanical properties to the standard ones, while all smaller specimens differed significantly. As the specimen size decreased, the measured strength and elongation values were diminished compared to the standard samples. Such a tendency is consistent with the findings of Molak et al. [39] or Kals et al. [42], and it is related to an increasing fraction of surface grains in smaller samples, which do not strengthen the material so effectively as internal grains. Thus, all other conditions of the 10CrMo9-10 steel were examined using miniaturized test specimens with a gauge length of 8 mm, showing the same results as the standard ones.

The comparison of representative stress–strain curves of the as-received, annealed and exploited 10CrMo9-10 steel is shown in Figure 4b, and the calculated mechanical parameters are summarized in Table 2. All annealed conditions exhibited an upper (*R_eH_*) and lower (*R_eL_*) yield strength, whereas the 0.2% offset yield strength (*R_p_*_0.2_) values were estimated for the as-received and exploited states. In general, the strength values decreased gradually with the prolongation of the annealing time, whereas the elongation values showed the opposite trend. The 10CrMo9-10 steel annealed for the longest period of 24 h exhibited *R_m_* of 467 ± 4 MPa and *A* of 37.3 ± 2.1% comparable to the exploited state (*R_m_* = 454 ± 4 MPa, *A* = 36.6 ± 1.3%), which is consistent with the hardness measurements presented in Figure 2b. At the same time, the exploited condition showed much lower *R_p_*_0.2_ = 218 ± 3 MPa than the annealed state after 24 h (*R_eH_* = 344 ± 7 MPa and *R_eL_* = 328 ± 4 MPa).

### 3.4. SPT Results

**Figure 5:**
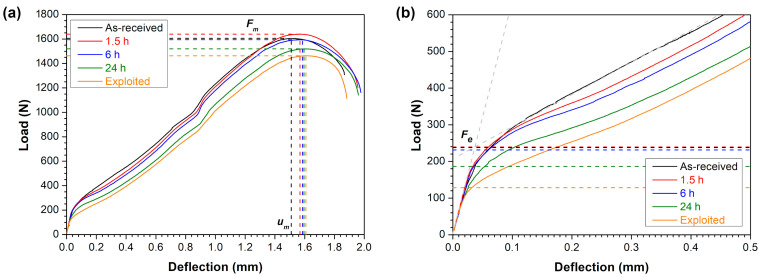
Representative load–deflection curves from SPTs of 10CrMo9-10 steel in as-received, annealed and exploited conditions: (**a**) overall view, (**b**) close-up of elastic bending regime.

In order to determine the linear correlations of the SPT results with the uniaxial tensile tests, the as-received, annealed and exploited 10CrMo9-10 steel was tested by using the SPT method. The representative load–deflection curves are shown in Figure 5, while Table 2 presents the average values of the elastic–plastic transition force (*F_e_*), maximum force (*F_m_*) and deflection at maximum force (*u_m_*). Four deformation stages, typically observed during SPT of a ductile material, can be distinguished in the obtained load–deflection curves, i.e., (I) elastic bending up to *F_e_* value, (II) plastic bending and (III) membrane stretching until reaching *F_m_* value and (IV) final plastic instability regime [20,43]. The average *F_m_* and *u_m_* for the as-received state was 1607 ± 28 N and 1.53 ± 0.02 mm. The annealing treatment for 1.5 h resulted in a slight increase in the *F_m_* value (1634 ± 25 N), but further exposition at 770 °C caused a gradual decrease in the maximum force to 1519 ± 25 N after 24 h. In turn, the deflection at maximum force was constantly increased due to the longer annealing treatment (as marked by dotted lines in Figure 5a). It is worth to mention that the changes in *F_m_* and *u_m_* values exhibited the same tendencies as the *R_m_* and *A_u_* during tensile tests. This implies that *F_m_* and *u_m_* values are sensitive to the observed microstructural changes in the same way as the *R_m_* and *A_u_*. The gradual reduction in *F_m_* value and strength of the 10CrMo9-10 steel after longer annealing periods resulted from the gradually decreased grain boundary density *S_V_* and carbide precipitates density *N_A_* (as shown in Figure 3). In turn, the higher content of equiaxed ferrite grains *V_V_* contributed to more uniform deformation and higher *u_m_* and *A_u_* values representing material ductility.

The close-up of the elastic bending regime presented in Figure 5b clearly shows that the transition forces *F_e_* for the as-received and annealed states for 1.5 and 6 h were very similar (in the range of 234–236 N), whereas they were drastically reduced for SPT specimens after annealing for 24 h (*F_e_* = 189 ± 5 N) and the exploitation period (*F_e_* = 135 ± 16 N). Such differences in the *F_e_* value are consistent with the tensile results, especially when the *R_eL_* values for the annealed conditions are considered, as recommended in the EN 10371:2021 standard. The *R_eL_* values for the annealed conditions gradually decreased from 422 ± 5 to 410 ± 7 and 328 ± 4 MPa after 1.5, 6 and 24 h at 770 °C, respectively, while the *F_e_* values were 236 ± 2, 234 ± 8 and 189 ± 5 N, respectively. In turn, the exploited condition clearly showed the lowest values of *F_e_* = 135 ± 16 N and *R_p_*_0.2_ = 218 ± 3 MPa. Such differences in the *F_e_* and *R_eL_* (or *R_p_*_0.2_) values can be linked to the gradually decreased grain boundary density *S_V_*, which contributed to less strengthening according to the well-known Hall–Petch relation [44,45].

**Figure 6:**
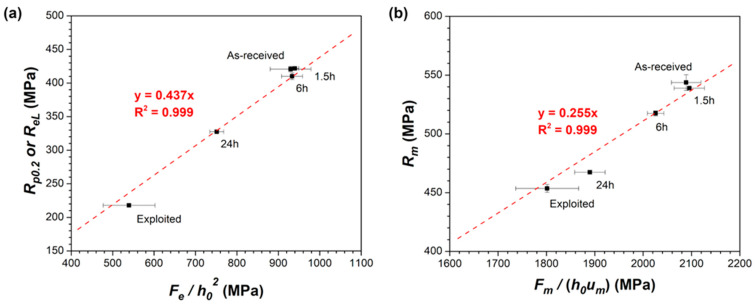
Linear correlations of SPT results with (**a**) yield strength and (**b**) ultimate tensile strength from uniaxial tensile tests.

Figure 6a presents the correlation function between *R_p_*_0.2_ (or *R_eL_*) from the uniaxial tensile test and *F_e_*/*h*_0_^2^ from the SPTs according to the Formula (1). The correlation factor *β_Rp_*_0.2_ was equal to 0.437. In turn, the correlation function between *R_m_* and *F_m_*/(*h*_0_ *u_m_)* according to the Formula (2) is presented in Figure 6b. The correlation factor *β_Rm_* was equal to 0.255. It should be mentioned that both calculated factors are similar to values reported in the literature. Table 3 summarizes the correlation factors with the geometry parameters of the used SPT setups. It has already been shown that the SPT correlation coefficients are more dependent on the SPT setup [31] rather than the investigated material [28,29,30]. Alstadt et al. [31] noticed for the T91 steel that the *β_Rp_*_0.2_ and *β_Rm_* factors may significantly differ from 0.42 to 0.63 and 0.19 to 0.26, respectively, depending on the used SPT setup, while the effect of the investigated material is less significant (*β_Rm_* = 0.254 ÷ 0.299 for P91, P92, Eurofer97, 22NiMoCr37 or 15Kh2MFA steel [30]). In addition, Garcia et al. [29] pointed out that the influence of specimen size and tested material may also vary depending on the applied method of strength correlation, but the empirical correlation equations used in the current SPT standard effectively minimize such effects. According to the EN 10371:2021 standard [27], for the standard geometry used in the current work (i.e., SPT specimen size Φ 8 mm × 0.5 mm, receiving hole diameter 4 mm, chamfer 0.2 × 45°, punch radius 1.25 mm), the coefficient *β_Rm_* should be in the range of 0.19 ≤ *β_Rm_* ≤ 0.32. The data presented in Table 3 also shows that the *β_Rm_* value for the standard geometry of the SPT setup is in the range from 0.21 to 0.278. Thus, the *β_Rm_* value of 0.255 obtained in the current study lies within the expectations. The estimated *β_Rp_*_0.2_ coefficient of 0.437 is also consistent with other literature references reporting values in the range of 0.42–0.49.

It should be highlighted that the obtained *β_Rp_*_0.2_ and *β_Rm_* factors allowed the calculation of the *R_p_*_0.2_ and *R_m_* of the exploited 10CrMo9-10 steel. Based on the SPT results, the values of *R_p_*_0.2_ = 236 ± 27 MPa and *R_m_* = 459 ± 17 MPa were estimated, which were close to *R_p_*_0.2_ = 218 ± 3 MPa and *R_m_* = 454 ± 4 MPa assessed in the uniaxial tensile tests. This result proves that the SPT method is a promising way of determining the changes in the mechanical properties of structural steels after long-term service at elevated temperature. Moreover, its predictive capability can be enhanced by incorporating machine learning methods [46,47]. Pan et al. [47] have investigated three different machine learning methods, such as back propagation (BP) neural network, radial basis function (RBF) network and random forest (RF) regression model. The prediction accuracy for pressure vessel steels was in the following order: BP > RBF > RF. The model proposed by Zhong et al. [46] exhibited the mean absolute percentage error in the SPT force prediction in the range of 0.5–2%. Finally, the overall assessment of the degradation state of the 10CrMo9-10 steel components can be improved by additional characterization techniques. The SPT method seems to be effective in the initial screening of mechanical performance (tensile strength, fracture toughness or creep properties), but it should to be supported by the precise characterization of carbide precipitates by transmission electron microscopy, segregation of impurities and possible depletion of alloying elements near grain boundaries by chemical analysis and high temperature corrosion and oxidation tests simulating the operational conditions of the 10CrMo9-10 steel.

## 4. Conclusions

The aim of this study was to develop a methodology for determining changes in the mechanical properties of 10CrMo9-10 steel using the SPT method, which was later successfully applied to estimate its mechanical performance after long-term exploitation for 280 000 h at 540 °C under pressure of 2.9 MPa. The main conclusions were drawn as follows:

1. The annealing of the as-received 10CrMo9-10 steel (*R_m_* = 544 MPa) at 770 °C for 1.5, 6 and 24 h allowed its strength to gradually decrease to *R_m_* = 539, 517 and 467 MPa, respectively, i.e., to the level similar to the exploited condition (*R_m_* = 454 MPa).
2. The lowered strength resulted from the gradually reduced grain boundary and carbide precipitates densities and the increased fraction of equiaxed ferrite grains after prolonged annealing.
3. The obtained SPT parameters for the as-received, annealed and exploited conditions (i.e., *F_e_*, *F_m_*, *u_m_*) exhibited the same tendencies as the *R_p_*_0.2_ (or *R_eL_*), *R_m_* and *A_u_* during tensile tests. The following correlation factors were determined *β_Rp_*_0.2_ = 0.437 and *β_Rm_* = 0.255 for the estimation of *R_p_*_0.2_ and *R_m_*, respectively.
4. Mechanical parameters of the exploited 10CrMo9-10 estimated based on the SPT results (*R_p_*_0.2_ = 236 ± 27 MPa and *R_m_* = 459 ± 17 MPa) were in a good agreement with those assessed during the uniaxial tensile tests (218 ± 3 MPa and *R_m_* = 454 ± 4 MPa).

## Figures and Tables

**Table 1:** Chemical composition of as-received and exploited 10CrMo9-10 steel.

Material	C	Si	Mn	Cr	Mo	Fe
As-received	0.12	0.37	0.42	2.00	0.90	Balanced
Exploited	0.16	0.44	0.61	2.51	0.98	Balanced
EN 10028-2	0.08–0.14	≤0.50	0.40–0.80	2.00–2.50	0.90–1.10	Balanced

**Table 2:** Mechanical properties of as-received, annealed and exploited 10CrMo9-10 steel (*R_p_*_0.2_—0.2% offset yield strength, *R_eH_*—upper yield strength, *R_eL_*—lower yield strength, *R_m_*—ultimate tensile strength, *A_u_*—uniform elongation, *A*—elongation at break, *F_e_*—elastic–plastic transition force in small punch test, *F_m_*—maximum force during small punch test, *u_m_*—deflection at maximum force).

Material	*R_p_*_0.2_ or *R_eH_*/*R_eL_* (MPa)	*R_m_* (MPa)	*A_u_* (%)	*A* (%)	*F_e_* (N)	*F_m_* (N)	*u_m_* (mm)
As-received (25 mm)	407 ± 11	538 ± 6	11.7 ± 0.2	32.1 ± 1.9	234 ± 13	1607 ± 28	1.53 ± 0.02
As-received (8 mm)	421 ± 6	544 ± 7	12.0 ± 0.1	31.7 ± 0.8			
As-received (4 mm)	398 ± 8	520 ± 7	11.8 ± 0.3	29.3 ± 1.3			
As-received (2 mm)	375 ± 9	484 ± 6	11.0 ± 0.5	27.8 ± 2.0			
Annealed 1.5 h	437 ± 14/422 ± 5	539 ± 4	16.1 ± 0.3	34.3 ± 1.5	236 ± 2	1634 ± 25	1.55 ± 0.01
Annealed 6 h	430 ± 10/410 ± 7	517 ± 4	17.4 ± 0.4	34.9 ± 1.3	234 ± 8	1599 ± 13	1.58 ± 0.01
Annealed 24 h	344 ± 7/328 ± 4	467 ± 4	20.3 ± 0.5	37.3 ± 2.1	189 ± 5	1519 ± 25	1.60 ± 0.01
Exploited	218 ± 3	454 ± 4	19.1 ± 0.3	36.6 ± 1.3	135 ± 16	1464 ± 47	1.62 ± 0.02

**Table 3:** Comparison of correlation factors *β_Rp_*_0.2_ and *β_Rm_* reported in the literature.

*β_Rp_*_0.2_	*β_Rm_*	Specimen Size (mm)	Receiving Hole Diameter (mm)	Chamfer Size or Edge Radius (mm)	Punch Radius (mm)	Materials Tested	Reference
0.437	0.255	Φ 8 × 0.5	4	0.2 × 45°	1.25	10CrMo9–10	This work
-	0.281	Φ 3 × 0.25	1.5	0.2	0.5	20MnMoNi55, CrMoV steel, SS304LN	[28]
0.442	0.277	10 × 10 × 0.5	4	0.2 × 45°	1.25	2.25Cr1Mo steels, dual phase steels, AISI 304, D2205, S460, API X70	[29]
-	0.278	Φ 8 × 0.5	4	0.2 × 45°	1.25	P91, P92, Eurofer97, 22NiMoCr37, 15Kh2MFA	[30]
0.63	0.26	3.5 × 3.5 × 0.25	1.75	0.25	0.5	T91	[31]
0.49	0.26	10 × 10 × 0.5	4	0.5	1.25	T91	[31]
0.48	0.21	Φ 8 × 0.5	4	0.2 × 45°	1.25	T91	[31]
0.42	0.19	Φ 3 × 0.25	1.5	0.2	0.5	T91	[31]

## Data Availability

The raw data supporting the conclusions of this article will be made available by the authors on request.
